# Breakthrough Tracheobronchial Aspergillosis Caused by *Aspergillus calidoustus* and *Aspergillus niger* in a Lung Transplant Recipient Despite Combined Antifungal Prophylaxis

**DOI:** 10.1155/crpu/7415627

**Published:** 2026-06-19

**Authors:** Carlos Enrique Calvache Benavides, Martha Daniela Rodríguez Cárdenas, Sergio Saul Sánchez Salazar, Rogelio de Jesús Treviño Rangel, Adrián Camacho Ortiz

**Affiliations:** ^1^ Center for Lung Transplantation and Advanced Respiratory Medicine (CETRA), Christus Muguerza Hospital Alta Especialidad, Monterrey, Nuevo Leon, Mexico; ^2^ Faculty of Medicine and University Hospital, Dr. José Eleuterio González, Autonomous University of Nuevo Leon, Monterrey, Nuevo Leon, Mexico, uanl.mx

**Keywords:** aspergillosis, fungal infection, isavuconazole, lung transplant

## Abstract

*Aspergillus niger* and *Aspergillus calidoustus* are relatively uncommon pathogens that could cause invasive disease, especially in immunocompromised patients. We present the case of a 61‐year‐old male with a history of idiopathic pulmonary fibrosis who was admitted to our hospital for a unilateral lung transplant that was performed without complications; despite voriconazole prophylaxis, he developed tracheobronchial aspergillosis, caused by *A. niger* and *A. calidoustus*—a species difficult to identify due to its close microbiological and genetic resemblance to other *Aspergillus* species. Molecular diagnostic methodologies are indispensable for the precise identification of cryptic *Aspergillus* species, a necessity emphatically demonstrated by this specific clinical presentation. The reliance upon these tools, in contrast to conventional phenotyping, is crucial for guiding appropriate and potentially life‐saving antifungal therapeutic interventions in critical scenarios like lung transplantation.

## 1. Introduction

Aspergillosis is a fungal infection that can affect both immunocompetent and immunocompromised patients, with a broad clinical spectrum ranging from asymptomatic manifestations, such as fungal sensitization or pulmonary granulomas, to highly lethal invasive infections [1]. These infections are particularly relevant in lung transplant recipients, in whom breakthrough infections despite antifungal prophylaxis represent a growing challenge and underscore the need for earlier and more accurate diagnosis [2–4].

## 2. Case Presentation

A 61‐year‐old man residing in Los Angeles, California, had a history of severe SARS‐CoV‐2 pneumonia and was subsequently diagnosed with idiopathic pulmonary fibrosis in the same year. The patient was a lifelong nonsmoker. During the pretransplant evaluation, an exhaustive etiological investigation for the fibrosis yielded negative results for autoimmune, environmental, or occupational triggers. He was initially treated with nintedanib but showed expected disease progression, eventually requiring home oxygen 2 years after diagnosis. The patient had no hospitalizations for severe exacerbations but experienced gradual deterioration in pulmonary function.

He was first admitted to our institution in February 2025 as part of the pre–lung transplant evaluation. Imaging studies, pulmonary function testing, and bronchoalveolar lavage (BAL) microbiology were all negative, including Gram stain, KOH, bacterial, and fungal cultures. Additionally, both latent and active tuberculosis were ruled out, including a negative QuantiFERON‐TB Gold assay. While serum 1,3‐beta‐D‐glucan was not routinely measured during the pretransplant workup, endemic fungal infections were ruled out via negative serologies (IgG and IgM) for *Coccidioides immitis* and *Histoplasma*. He was discharged and readmitted in June for a planned right single‐lung transplant, which proceeded without complications. Postoperatively, he was managed in the intensive care unit with invasive monitoring and advanced ventilatory support.

In accordance with our center′s protocol, perioperative induction immunosuppression (e.g., basiliximab or antithymocyte globulin) was omitted to avoid overimmunosuppression, as there is currently no universal consensus mandating its routine use in lung transplantation. Maintenance immunosuppressive therapy included methylprednisolone 125 mg BID, tacrolimus 1.5 mg BID, and mycophenolate mofetil 1000 mg BID. Antimicrobial prophylaxis consisted of trimethoprim–sulfamethoxazole 800/160 mg QD, nebulized amikacin 50 mg in 3 mL saline BID, and antifungal prophylaxis with voriconazole 400 mg BID for two doses, followed by 200 mg BID.

**Figure 1:**
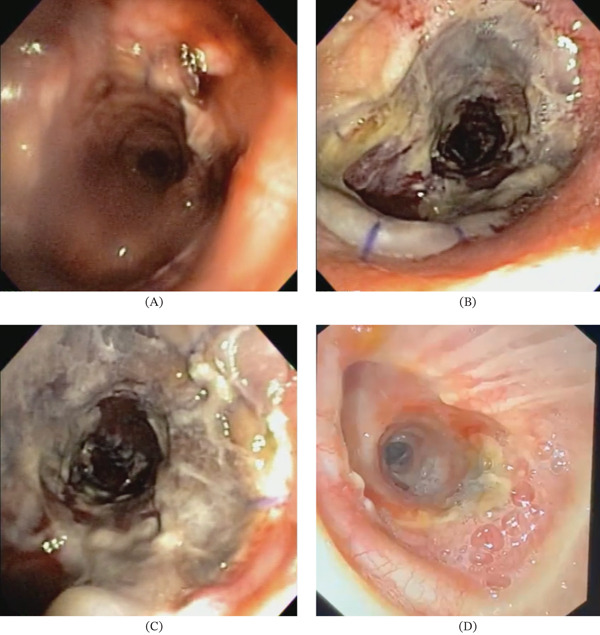
(A) Bronchial anastomosis on POD 5 during voriconazole prophylaxis. (B) Bronchial anastomosis on POD 8 coinciding with the initiation of isavuconazole and amphotericin B. (C) Bronchial anastomosis on POD 12, after 4 days of isavuconazole and amphotericin B treatment. (D) Bronchial anastomosis on POD 38 after 26 days of isavuconazole and amphotericin B treatment.

Respiratory support was gradually reduced to a high‐flow nasal cannula. The first postoperative bronchoscopy, performed on POD (postoperative day) 2, showed surgical changes with blood remnants at the anastomotic site (Figure 1A). Samples were obtained for fungal culture. Results are summarized in Table 1.

**Table 1:** Results of galactomannan testing and fungal cultures.

Postoperative day	Sample type	Galactomannan result^a^	Culture result
38	BAL	0.81	Negative at 30 days
21	BAL	12.46	*Aspergillus calidoustus*
12	BAL	7.04	*Aspergillus calidoustus*
8	Tracheal aspirate	2.50	*Aspergillus niger*
5	BAL	1.74	*Aspergillus niger*
0	BAL	Not performed	Negative at 30 days
−101	Sputum	Not performed	Negative at 30 days

^a^Optical density index (ODI).

Although the patient′s course was initially favorable, the initial BAL culture reported hyphal growth. A second bronchoscopy performed 4 days later revealed erythematous, friable mucosa with purulent plaques at the anastomosis (Figure 1B). New samples were obtained for fungal culture and galactomannan testing. Based on these findings, antifungal management was switched from prophylactic voriconazole to therapeutic isavuconazole 200 mg IV TID for six doses, followed by a 200 mg QD dose, along with nebulized Amphotericin B BID. Galactomannan from POD 5 BAL was positive at 1.74 ODI (optical density index).

The patient remained clinically stable, with no signs of deterioration. Eight days posttransplant, despite antifungal therapy, BAL persisted with hyphal growth. A third bronchoscopy revealed purulent secretions, necrotic mucosa, hyperemia, and granulation tissue (Figure 1C). Galactomannan increased to 2.5 ODI on POD 8. Given these findings, a thoracic computed tomography (CT) scan was performed to assess for potential parenchymal or angioinvasive involvement, which showed adequate right lung expansion with no abnormal opacifications or consolidations. The first fungal culture identified *Aspergillus niger*; however, its specific susceptibility results were deemed not clinically pivotal for the prolonged management of this case and were congruent with expected wild‐type susceptibility. Antifungal susceptibility testing was performed using the broth microdilution method in accordance with the CLSI M38 guidelines. Consistent with this method, minimum inhibitory concentrations (MICs) were determined visually after 24–48 h, while minimum effective concentrations (MECs) were established for echinocandins. The specific MICs for the *Aspergillus calidoustus* isolate on POD 12 were 0.25 *μ*g/mL for voriconazole, 0.5 *μ*g/mL for isavuconazole, and 0.062 *μ*g/mL for Amphotericin B. Oral isavuconazole, 200 mg QD, was continued. The patient′s condition improved, and supplemental oxygen was discontinued.

Subsequent bronchoscopies revealed progressive improvement, with cultures repeatedly isolating the same *Aspergillus* species. On POD 12, BAL galactomannan rose to 7.04 ODI; however, given the favorable clinical evolution, he was discharged for outpatient follow‐up.

A bronchoscopy on POD 18 showed marked improvement in mucosal erythema and necrosis. However, purulent debris persisted, with a galactomannan of 12.46 ODI, and culture remained positive for *A. calidoustus*.

Given the susceptibility profile and clinical and bronchoscopic improvement, therapy with isavuconazole and nebulized Amphotericin B was maintained. On subsequent follow‐up (POD 38), the bronchoscopy revealed no evidence of infection at the anastomosis site (Figure 1D), BAL galactomannan levels dropped to 0.81 ODI, and fungal cultures remained negative after 30 days. The infection was classified as breakthrough tracheobronchial aspergillosis due to *A. niger* and *A. calidoustus* despite voriconazole prophylaxis.

At the 10‐month follow‐up evaluation, the patient remains in excellent clinical condition and does not require supplemental oxygen support. Notably, he has not required hospital readmission for pulmonary deterioration or any infectious etiology. As part of the surveillance protocol, a follow‐up thoracic CT scan showed no evidence of consolidations, infiltrative patterns, or secondary lymphadenopathy, ruling out signs of chronic pulmonary infection. His last surveillance bronchoscopy confirmed sustained fungal clearance with a BAL galactomannan of 0.8 ODI and negative cultures.

## 3. Discussion

This case underscores the increasing diagnostic and therapeutic challenges posed by invasive fungal infections in immunocompromised patients, especially in solid organ transplant recipients. Tracheobronchial and invasive aspergillosis remain significant causes of morbidity and mortality in this population [1]. Accurate species identification and antifungal susceptibility testing are essential to ensure adequate treatment and prevent adverse outcomes.

This patient, with no prior evidence of *Aspergillus* colonization, developed an early tracheobronchial infection at the anastomotic site with initial isolation of *A. niger*. This presentation is a well‐recognized manifestation of aspergillosis in lung transplant recipients, in whom fungal hyphae may directly invade anastomotic tissue [1]. *Aspergillus niger* is among the most frequently isolated *Aspergillus* species in human infection. The FUNGAE‐IFI study reported an incidence of 4.1%, ranking fifth in frequency [2], and the Spanish FILPOP study reported *A. niger* in 6.5% of isolates [3]. *Aspergillus niger* generally remains susceptible to azoles, Amphotericin B, and echinocandins. However, resistant strains have been reported [2].

In this case, subsequent identification of *A. calidoustus* at the same site, along with increasing galactomannan levels despite voriconazole prophylaxis, confirmed a breakthrough infection. Given the limitations of phenotypic identification for cryptic species, the definitive species confirmation of *A. niger* and *A. calidoustus* was achieved through molecular techniques. After preliminary morphological assessment, genomic DNA was extracted, and three genetic loci (the internal transcribed spacer region, a fragment of the *β*‐tubulin gene, and a portion of the calmodulin gene) were amplified by PCR and analyzed via Sanger sequencing. Morphologically, *A. calidoustus* differs from common species like *Aspergillus fumigatus* or *A. niger* by its characteristic drab olive to brown conidia and the production of Hülle cells, though definitive differentiation relies on these specific molecular targets. This is clinically relevant not only because of the rarity of *A. calidoustus* isolation but also due to its antifungal resistance profile and the potential for coinfection with distinct *Aspergillus* species. Selective pressure from prolonged azole use has contributed to the emergence of “cryptic” *Aspergillus* species that are intrinsically resistant to azoles [4]. The therapeutic approach for *A. calidoustus* significantly differs from other common *Aspergillus* species. While *A. niger* typically remains highly susceptible to triazoles, *A. calidoustus* is intrinsically resistant to multiple azoles, often rendering voriconazole or posaconazole ineffective. Consequently, the treatment of choice shifts toward Amphotericin B, newer echinocandins, or combination therapies (e.g., isavuconazole with terbinafine) [3, 4].

*Aspergillus calidoustus* has emerged as a pathogen particularly in immunocompromised hosts, especially hematopoietic stem cell transplant recipients [5] and, to a lesser extent, solid organ transplant patients [6]. First recognized in 2008 (formerly classified within the *Ustus* complex), *A. calidoustus* has been described as an emerging pathogen among lung transplant recipients receiving long‐term azole prophylaxis, particularly voriconazole. Such prophylaxis suppresses *A. fumigatus* while promoting the growth of resistant cryptic species, such as *A. calidoustus*. Duration of azole exposure is the only significant risk factor for infection identified in case–control studies [4].

Accurate identification of *A. calidoustus* is essential, as phenotypic methods are often insufficient. Molecular tools, including sequencing of *β*‐tubulin, calmodulin, and actin genes, are necessary for definitive differentiation [7, 8].

Cryptic *Aspergillus* species account for 10%–19% of clinical isolates [6]. The FILPOP study found 15% cryptic species among 323 isolates [3], while FUNGAE‐IFI reported 12.5% [2]. Clinically, this is significant given *A. calidoustus*′s high azole MICs (> 32 mg/L by *E*‐test), indicating reduced or absent susceptibility [2]. While Amphotericin B, newer echinocandins (e.g., rezafungin), and terbinafine demonstrate better in vitro activity [3, 6, 9, 10], their clinical use is limited by toxicity or drug interactions. The therapeutic approach for *A. calidoustus* significantly differs from other common *Aspergillus* species. Recent data from European multicenter registries highlight that *A. calidoustus* consistently exhibits high in vitro MICs to multiple azole drugs, confirming its intrinsically resistant profile [11]. The specific MICs observed in our patient (voriconazole 0.25 *μ*g/mL and isavuconazole 0.5 *μ*g/mL) were unusually low for this cryptic species, yet the clinical breakthrough underscores the limitations of azole monotherapy in this setting. For typical multidrug‐resistant isolates, the treatment of choice shifts toward Amphotericin B or newer echinocandins. Furthermore, recent studies have demonstrated a potent synergistic interaction between azoles (e.g., voriconazole or isavuconazole) and terbinafine specifically against *A. calidoustus*, offering a promising combination strategy to decrease inhibitory dosages and overcome resistance in refractory clinical scenarios [12].

Meanwhile, isavuconazole remains a first‐line therapy for invasive aspergillosis and has shown synergistic in vitro activity when combined with terbinafine [13]. This contrasts with the high mortality (up to 66%) typically associated with *A. calidoustus* infections [6].

Breakthrough fungal infections under antifungal prophylaxis represent a major clinical concern due to their high mortality risk [1]. Biomarker monitoring (e.g., galactomannan) should be complemented with culture, microscopy, molecular identification, and susceptibility testing [7, 8], enabling the detection of uncommon pathogens and guiding optimal antifungal therapy [14].

In conclusion, the emergence of cryptic species such as *A. calidoustus* in lung transplant recipients receiving azole prophylaxis demands a high clinical index of suspicion. The take‐home message for clinical practice is twofold: First, molecular identification is mandatory when atypical or breakthrough fungal infections occur; second, empirical shifting to nonazole therapies, such as Amphotericin B, has to be considered based on clinical settings until specific susceptibility profiles are established, given the intrinsic azole resistance of these emerging pathogens.

**Nomenclature**
BAL: bronchoalveolar lavage
mg: milligram
BID: bis in die (twice daily)
QD: quaque die (once daily)
ml: milliliter
POD: postoperative day
IV: intravenous
ODI: optical density index
MIC: minimum inhibitory concentration

## Funding

No funding was received for this manuscript.

## Conflicts of Interest

The authors declare no conflicts of interest.

## Data Availability

The data that support the findings of this study are available from the corresponding author upon reasonable request.
